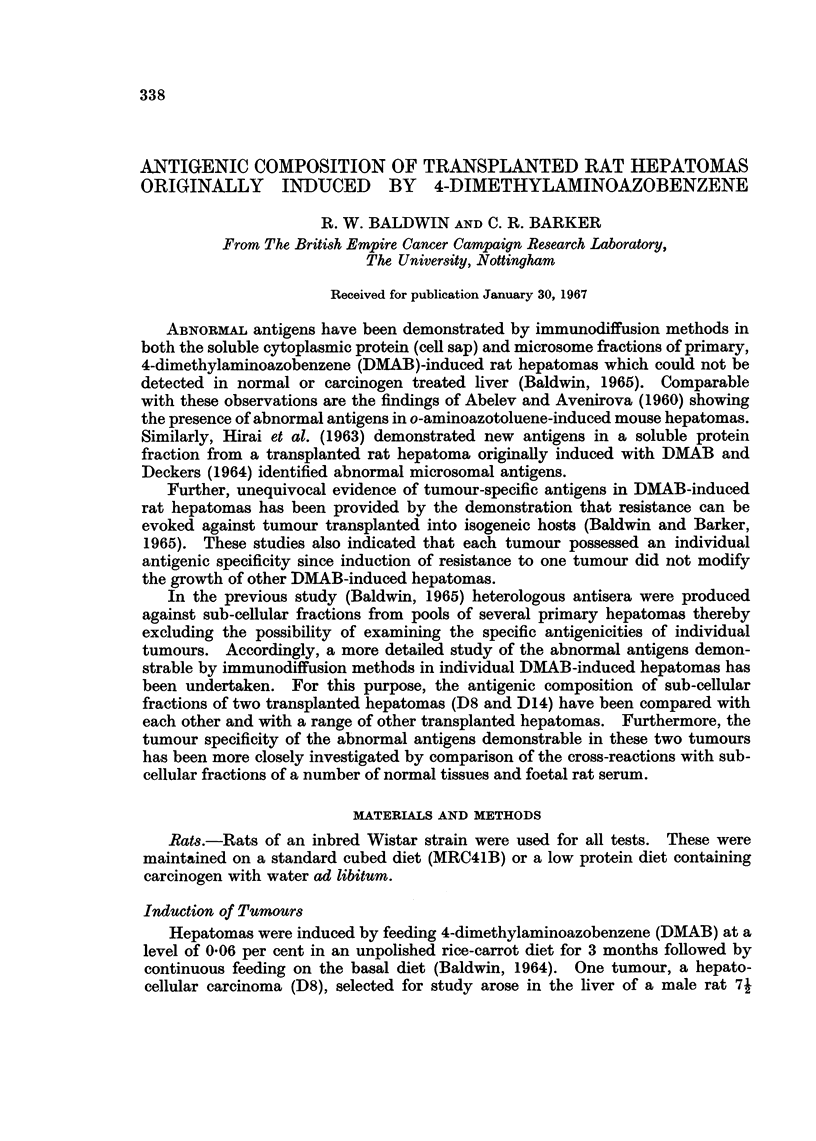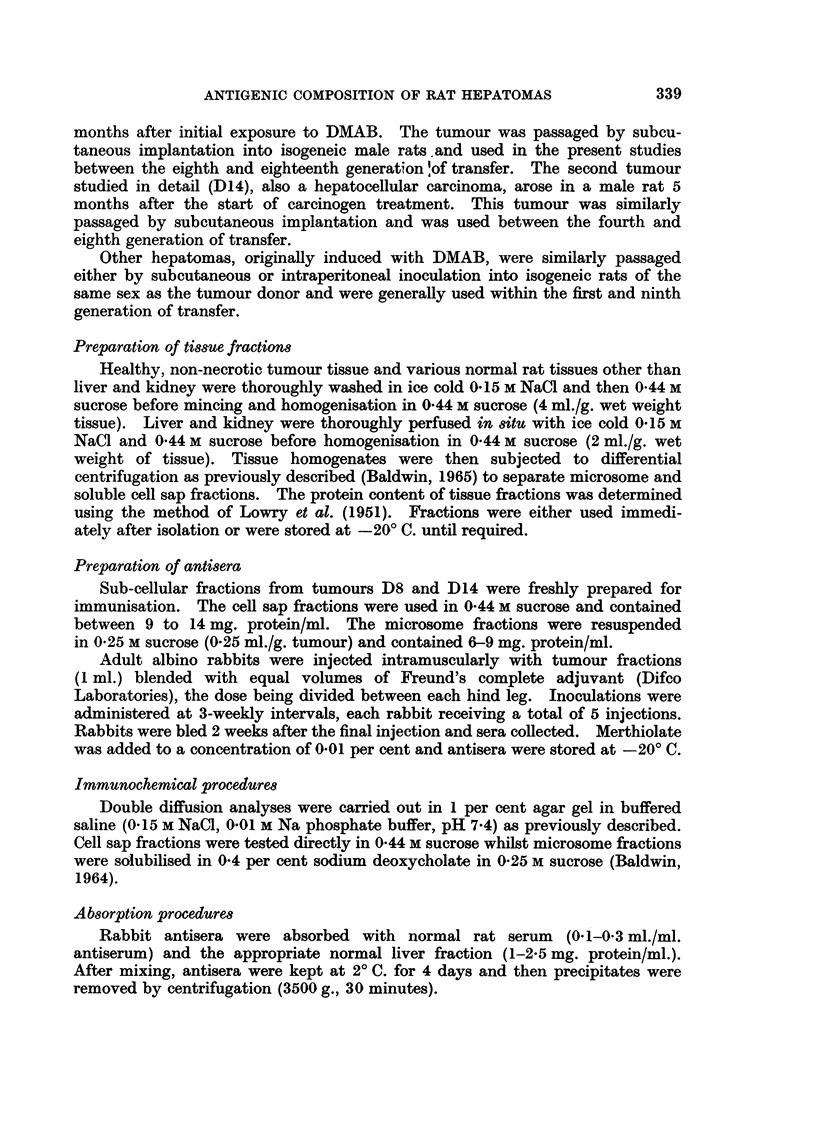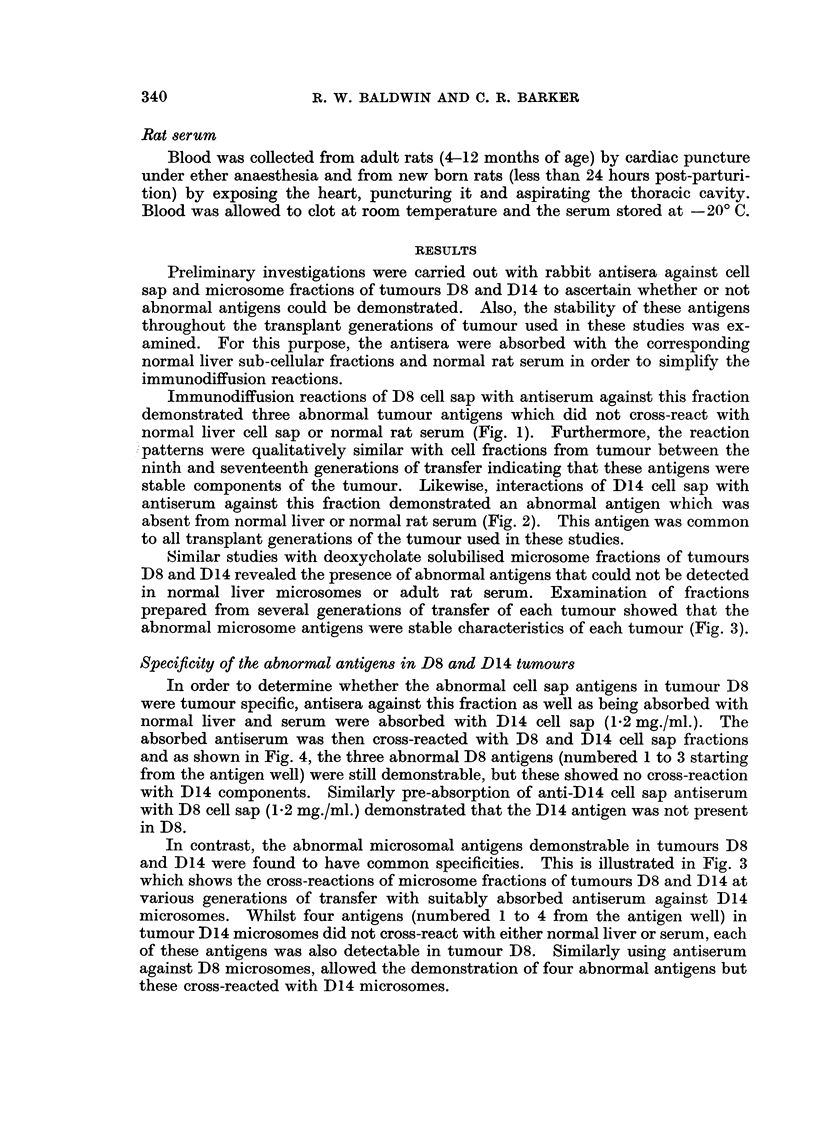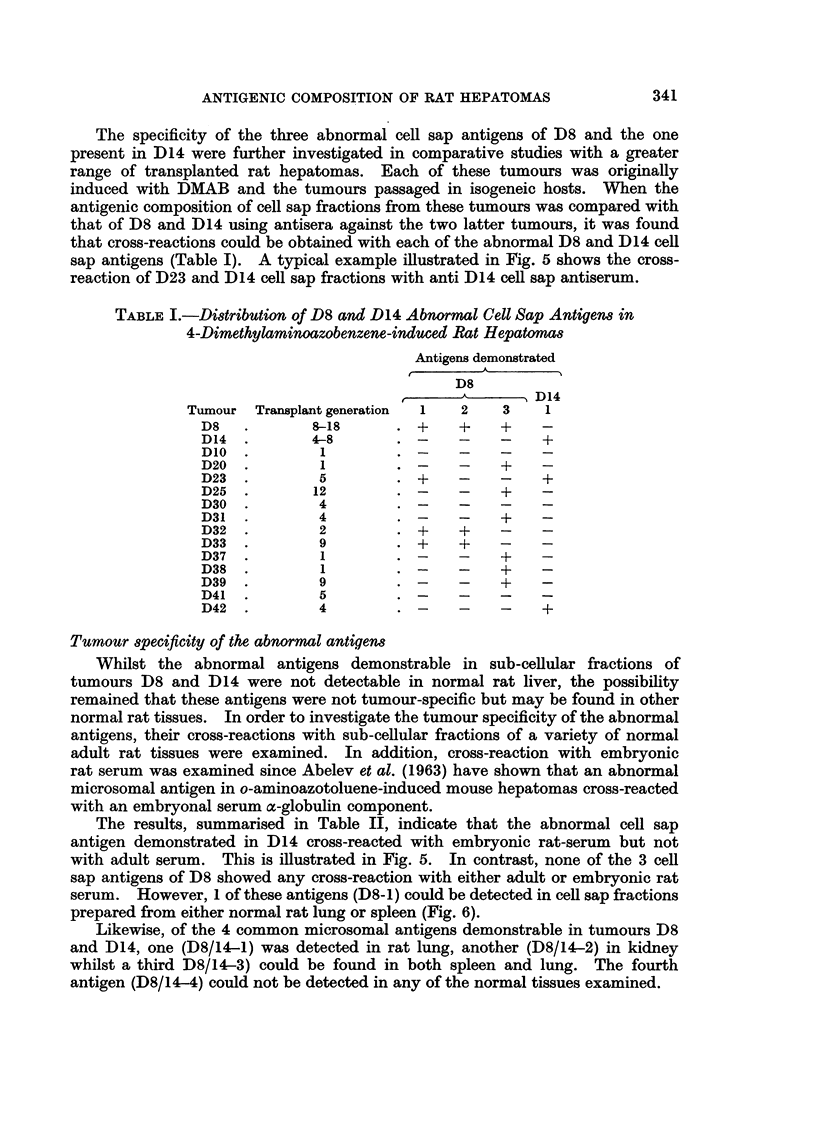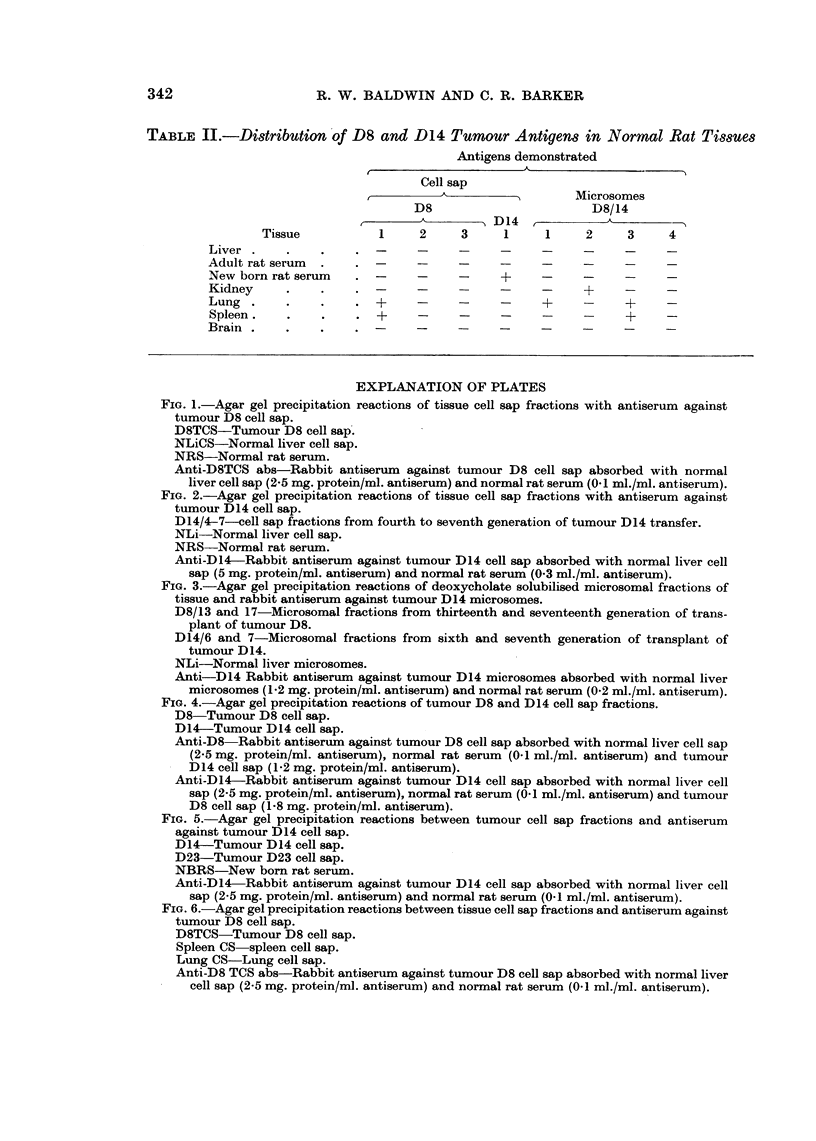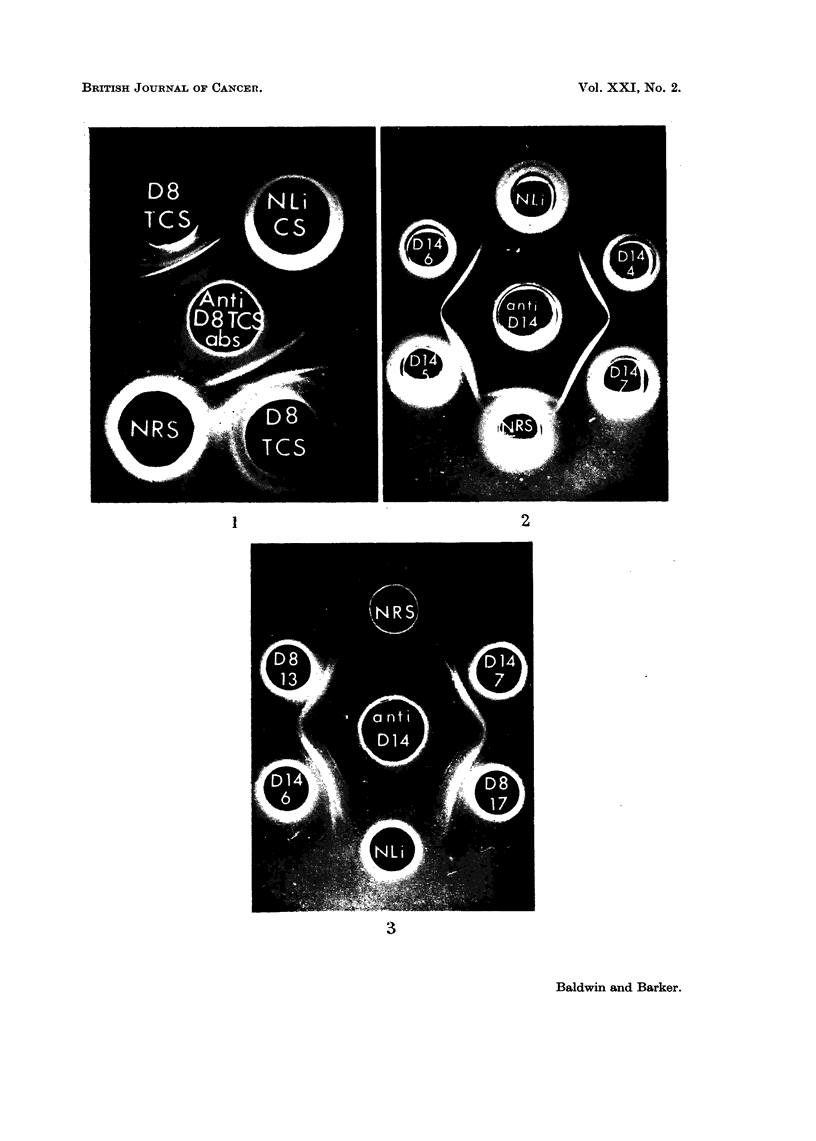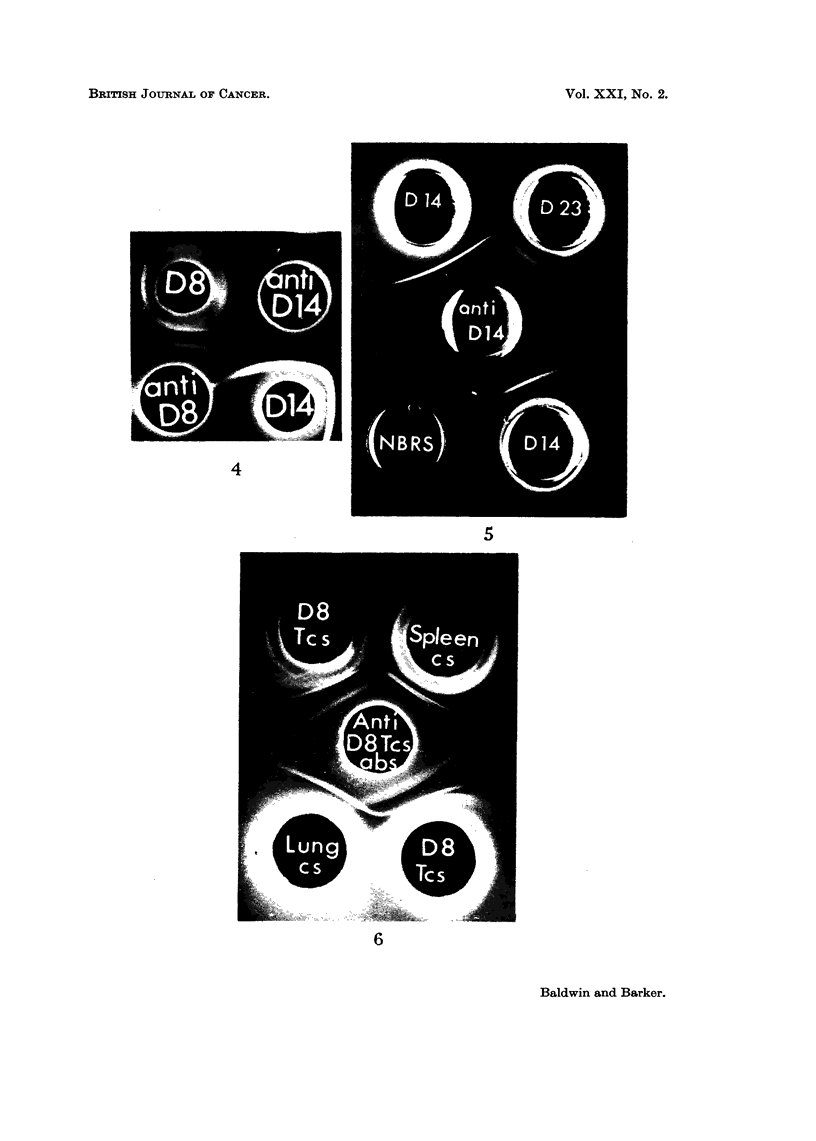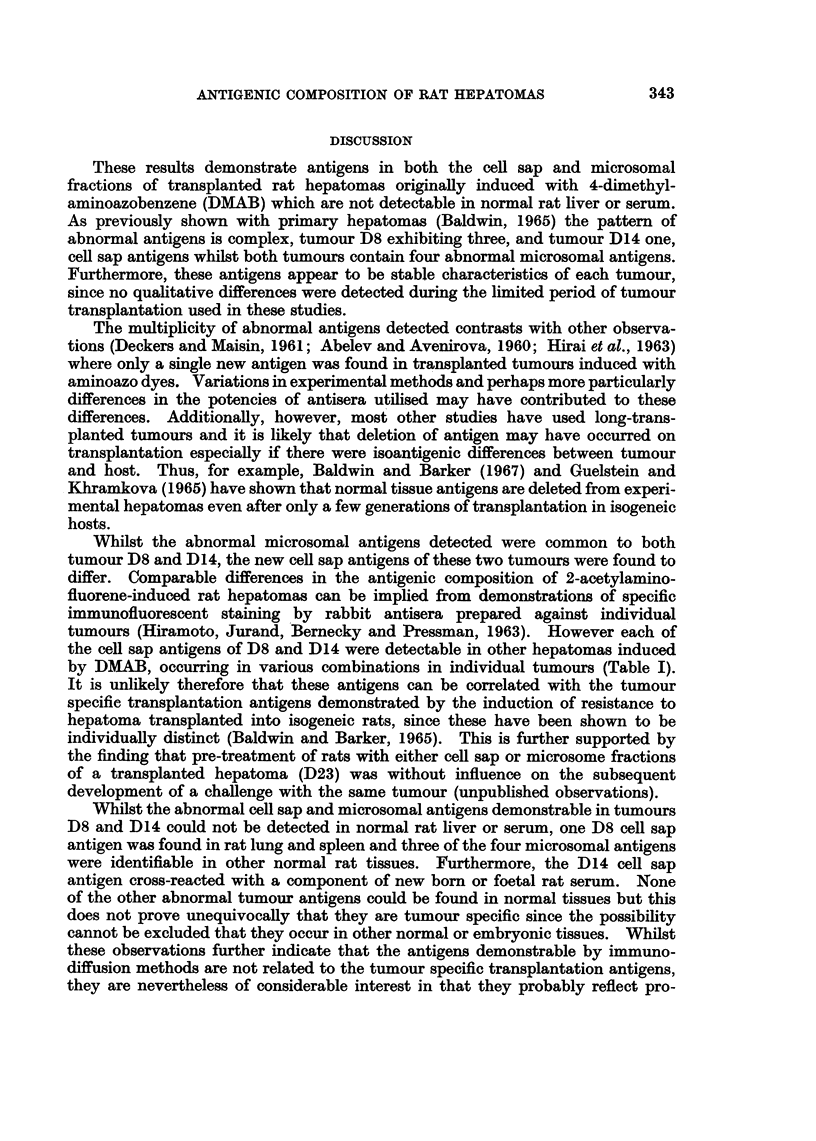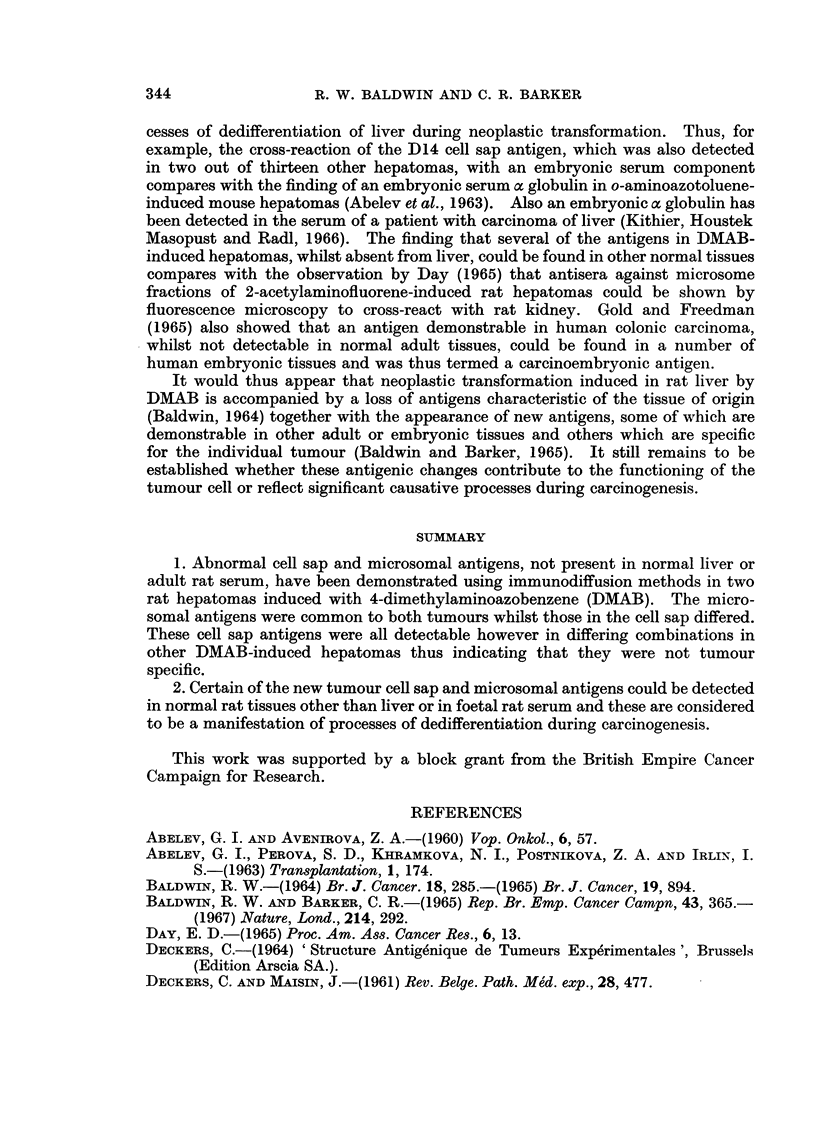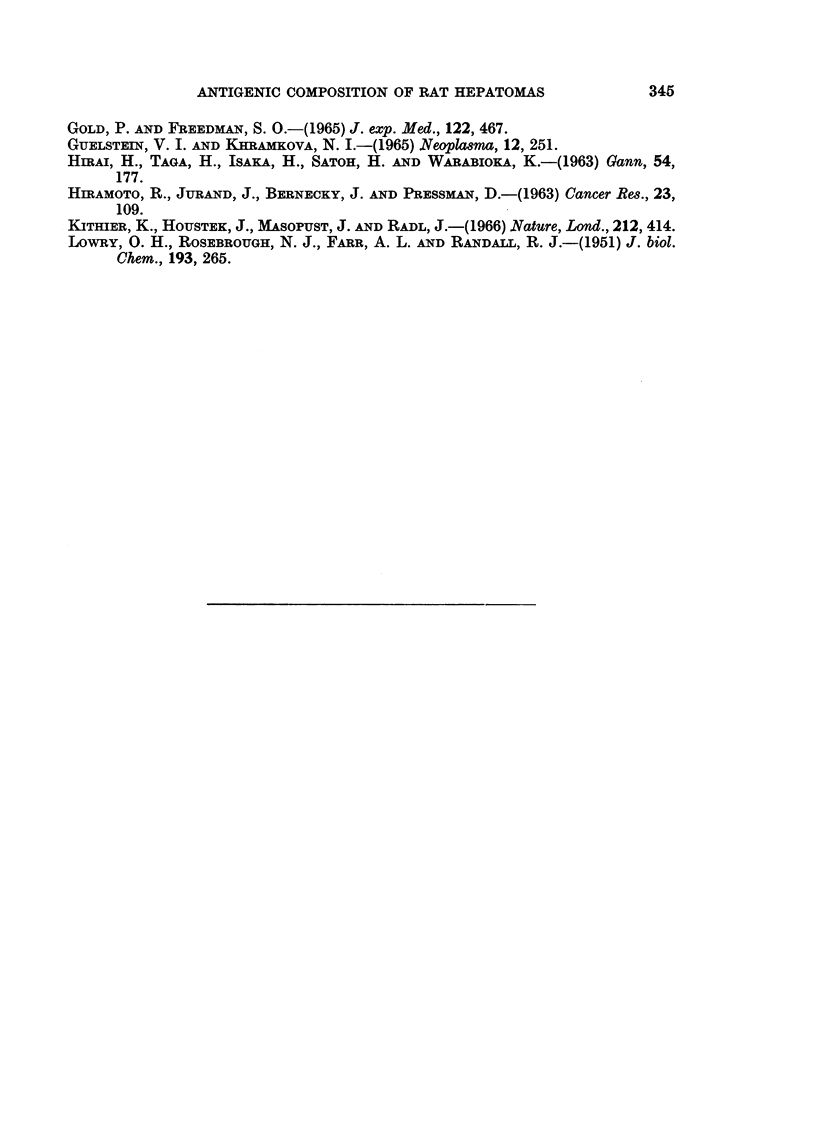# Antigenic composition of transplanted rat hepatomas originally induced by 4-dimethylaminoazobenzene.

**DOI:** 10.1038/bjc.1967.38

**Published:** 1967-06

**Authors:** R. W. Baldwin, C. R. Barker

## Abstract

**Images:**


### ANTIGENIC COMPOSITION OF TRANSPLANTED RAT HEPATOMAS ORIGINALLY INDUCED BY 4-DIMETHYLAMINOAZOBENZENE

R. W. BALDWIN AND C. R. BARKER

*From The British Empire Cancer Campaign Research Laboratory, The University, Nottingham*

Received for publication January 30, 1967

ABNORMAL antigens have been demonstrated by immunodiffusion methods in both the soluble cytoplasmic protein (cell sap) and microsome fractions of primary, 4-dimethylaminoazobenzene (DMAB)-induced rat hepatomas which could not be detected in normal or carcinogen treated liver (Baldwin, 1965). Comparable with these observations are the findings of Abelev and Avenirova (1960) showing the presence of abnormal antigens in *o*-aminoazotoluene-induced mouse hepatomas. Similarly, Hirai *et al.* (1963) demonstrated new antigens in a soluble protein fraction from a transplanted rat hepatoma originally induced with DMAB and Deckers (1964) identified abnormal microsomal antigens.

Further, unequivocal evidence of tumour-specific antigens in DMAB-induced rat hepatomas has been provided by the demonstration that resistance can be evoked against tumour transplanted into isogeneic hosts (Baldwin and Barker, 1965). These studies also indicated that each tumour possessed an individual antigenic specificity since induction of resistance to one tumour did not modify the growth of other DMAB-induced hepatomas.

In the previous study (Baldwin, 1965) heterologous antisera were produced against sub-cellular fractions from pools of several primary hepatomas thereby excluding the possibility of examining the specific antigenicities of individual tumours. Accordingly, a more detailed study of the abnormal antigens demonstrable by immunodiffusion methods in individual DMAB-induced hepatomas has been undertaken. For this purpose, the antigenic composition of sub-cellular fractions of two transplanted hepatomas (D8 and D14) have been compared with each other and with a range of other transplanted hepatomas. Furthermore, the tumour specificity of the abnormal antigens demonstrable in these two tumours has been more closely investigated by comparison of the cross-reactions with sub-cellular fractions of a number of normal tissues and foetal rat serum.

### MATERIALS AND METHODS

*Rats*.—Rats of an inbred Wistar strain were used for all tests. These were maintained on a standard cubed diet (MRC41B) or a low protein diet containing carcinogen with water *ad libitum*.

### *Induction of Tumours*

Hepatomas were induced by feeding 4-dimethylaminoazobenzene (DMAB) at a level of 0·06 per cent in an unpolished rice-carrot diet for 3 months followed by continuous feeding on the basal diet (Baldwin, 1964). One tumour, a hepatocellular carcinoma (D8), selected for study arose in the liver of a male rat 7½

months after initial exposure to DMAB. The tumour was passaged by subcutaneous implantation into isogeneic male rats and used in the present studies between the eighth and eighteenth generation of transfer. The second tumour studied in detail (D14), also a hepatocellular carcinoma, arose in a male rat 5 months after the start of carcinogen treatment. This tumour was similarly passaged by subcutaneous implantation and was used between the fourth and eighth generation of transfer.

Other hepatomas, originally induced with DMAB, were similarly passaged either by subcutaneous or intraperitoneal inoculation into isogeneic rats of the same sex as the tumour donor and were generally used within the first and ninth generation of transfer.

*Preparation of tissue fractions*

Healthy, non-necrotic tumour tissue and various normal rat tissues other than liver and kidney were thoroughly washed in ice cold 0·15 M NaCl and then 0·44 M sucrose before mincing and homogenisation in 0·44 M sucrose (4 ml./g. wet weight tissue). Liver and kidney were thoroughly perfused *in situ* with ice cold 0·15 M NaCl and 0·44 M sucrose before homogenisation in 0·44 M sucrose (2 ml./g. wet weight of tissue). Tissue homogenates were then subjected to differential centrifugation as previously described (Baldwin, 1965) to separate microsome and soluble cell sap fractions. The protein content of tissue fractions was determined using the method of Lowry *et al.* (1951). Fractions were either used immediately after isolation or were stored at —20° C. until required.

*Preparation of antisera*

Sub-cellular fractions from tumours D8 and D14 were freshly prepared for immunisation. The cell sap fractions were used in 0·44 M sucrose and contained between 9 to 14 mg. protein/ml. The microsome fractions were resuspended in 0·25 M sucrose (0·25 ml./g. tumour) and contained 6–9 mg. protein/ml.

Adult albino rabbits were injected intramuscularly with tumour fractions (1 ml.) blended with equal volumes of Freund's complete adjuvant (Difco Laboratories), the dose being divided between each hind leg. Inoculations were administered at 3-weekly intervals, each rabbit receiving a total of 5 injections. Rabbits were bled 2 weeks after the final injection and sera collected. Merthiolate was added to a concentration of 0·01 per cent and antisera were stored at —20° C.

*Immunochemical procedures*

Double diffusion analyses were carried out in 1 per cent agar gel in buffered saline (0·15 M NaCl, 0·01 M Na phosphate buffer, pH 7·4) as previously described. Cell sap fractions were tested directly in 0·44 M sucrose whilst microsome fractions were solubilised in 0·4 per cent sodium deoxycholate in 0·25 M sucrose (Baldwin, 1964).

*Absorption procedures*

Rabbit antisera were absorbed with normal rat serum (0·1–0·3 ml./ml. antiserum) and the appropriate normal liver fraction (1–2·5 mg. protein/ml.). After mixing, antisera were kept at 2° C. for 4 days and then precipitates were removed by centrifugation (3500 g., 30 minutes).

*Rat serum*

Blood was collected from adult rats (4–12 months of age) by cardiac puncture under ether anaesthesia and from new born rats (less than 24 hours post-parturition) by exposing the heart, puncturing it and aspirating the thoracic cavity. Blood was allowed to clot at room temperature and the serum stored at $-20°$ C.

### RESULTS

Preliminary investigations were carried out with rabbit antisera against cell sap and microsome fractions of tumours D8 and D14 to ascertain whether or not abnormal antigens could be demonstrated. Also, the stability of these antigens throughout the transplant generations of tumour used in these studies was examined. For this purpose, the antisera were absorbed with the corresponding normal liver sub-cellular fractions and normal rat serum in order to simplify the immunodiffusion reactions.

Immunodiffusion reactions of D8 cell sap with antiserum against this fraction demonstrated three abnormal tumour antigens which did not cross-react with normal liver cell sap or normal rat serum (Fig. 1). Furthermore, the reaction patterns were qualitatively similar with cell fractions from tumour between the ninth and seventeenth generations of transfer indicating that these antigens were stable components of the tumour. Likewise, interactions of D14 cell sap with antiserum against this fraction demonstrated an abnormal antigen which was absent from normal liver or normal rat serum (Fig. 2). This antigen was common to all transplant generations of the tumour used in these studies.

Similar studies with deoxycholate solubilised microsome fractions of tumours D8 and D14 revealed the presence of abnormal antigens that could not be detected in normal liver microsomes or adult rat serum. Examination of fractions prepared from several generations of transfer of each tumour showed that the abnormal microsome antigens were stable characteristics of each tumour (Fig. 3).

*Specificity of the abnormal antigens in D8 and D14 tumours*

In order to determine whether the abnormal cell sap antigens in tumour D8 were tumour specific, antisera against this fraction as well as being absorbed with normal liver and serum were absorbed with D14 cell sap (1·2 mg./ml.). The absorbed antiserum was then cross-reacted with D8 and D14 cell sap fractions and as shown in Fig. 4, the three abnormal D8 antigens (numbered 1 to 3 starting from the antigen well) were still demonstrable, but these showed no cross-reaction with D14 components. Similarly pre-absorption of anti-D14 cell sap antiserum with D8 cell sap (1·2 mg./ml.) demonstrated that the D14 antigen was not present in D8.

In contrast, the abnormal microsomal antigens demonstrable in tumours D8 and D14 were found to have common specificities. This is illustrated in Fig. 3 which shows the cross-reactions of microsome fractions of tumours D8 and D14 at various generations of transfer with suitably absorbed antiserum against D14 microsomes. Whilst four antigens (numbered 1 to 4 from the antigen well) in tumour D14 microsomes did not cross-react with either normal liver or serum, each of these antigens was also detectable in tumour D8. Similarly using antiserum against D8 microsomes, allowed the demonstration of four abnormal antigens but these cross-reacted with D14 microsomes.

The specificity of the three abnormal cell sap antigens of D8 and the one present in D14 were further investigated in comparative studies with a greater range of transplanted rat hepatomas. Each of these tumours was originally induced with DMAB and the tumours passaged in isogeneic hosts. When the antigenic composition of cell sap fractions from these tumours was compared with that of D8 and D14 using antisera against the two latter tumours, it was found that cross-reactions could be obtained with each of the abnormal D8 and D14 cell sap antigens (Table I). A typical example illustrated in Fig. 5 shows the cross-reaction of D23 and D14 cell sap fractions with anti D14 cell sap antiserum.

TABLE I.—*Distribution of D8 and D14 Abnormal Cell Sap Antigens in 4-Dimethylaminoazobenzene-induced Rat Hepatomas*

| | | Antigens demonstrated | | | |
|---|---|---|---|---|---|
| | | D8 | | | D14 |
| Tumour | Transplant generation | 1 | 2 | 3 | 1 |
| D8 | 8–18 | + | + | + | − |
| D14 | 4–8 | − | − | − | + |
| D10 | 1 | − | − | − | − |
| D20 | 1 | − | − | + | − |
| D23 | 5 | + | − | − | + |
| D25 | 12 | − | − | + | − |
| D30 | 4 | − | − | − | − |
| D31 | 4 | − | − | + | − |
| D32 | 2 | + | + | − | − |
| D33 | 9 | + | + | − | − |
| D37 | 1 | − | − | + | − |
| D38 | 1 | − | − | + | − |
| D39 | 9 | − | − | + | − |
| D41 | 5 | − | − | − | − |
| D42 | 4 | − | − | − | + |

*Tumour specificity of the abnormal antigens*

Whilst the abnormal antigens demonstrable in sub-cellular fractions of tumours D8 and D14 were not detectable in normal rat liver, the possibility remained that these antigens were not tumour-specific but may be found in other normal rat tissues. In order to investigate the tumour specificity of the abnormal antigens, their cross-reactions with sub-cellular fractions of a variety of normal adult rat tissues were examined. In addition, cross-reaction with embryonic rat serum was examined since Abelev *et al.* (1963) have shown that an abnormal microsomal antigen in *o*-aminoazotoluene-induced mouse hepatomas cross-reacted with an embryonal serum $\alpha$-globulin component.

The results, summarised in Table II, indicate that the abnormal cell sap antigen demonstrated in D14 cross-reacted with embryonic rat-serum but not with adult serum. This is illustrated in Fig. 5. In contrast, none of the 3 cell sap antigens of D8 showed any cross-reaction with either adult or embryonic rat serum. However, 1 of these antigens (D8-1) could be detected in cell sap fractions prepared from either normal rat lung or spleen (Fig. 6).

Likewise, of the 4 common microsomal antigens demonstrable in tumours D8 and D14, one (D8/14–1) was detected in rat lung, another (D8/14–2) in kidney whilst a third D8/14–3) could be found in both spleen and lung. The fourth antigen (D8/14–4) could not be detected in any of the normal tissues examined.

TABLE II.—*Distribution of D8 and D14 Tumour Antigens in Normal Rat Tissues*

| | Antigens demonstrated | | | | | | | |
|---|---|---|---|---|---|---|---|---|
| | Cell sap | | | | Microsomes D8/14 | | | |
| | D8 | | | D14 | | | | |
| Tissue | 1 | 2 | 3 | 1 | 1 | 2 | 3 | 4 |
| Liver | — | — | — | — | — | — | — | — |
| Adult rat serum | — | — | — | — | — | — | — | — |
| New born rat serum | — | — | — | + | — | — | — | — |
| Kidney | — | — | — | — | — | + | — | — |
| Lung | + | — | — | — | + | — | + | — |
| Spleen | + | — | — | — | — | — | + | — |
| Brain | — | — | — | — | — | — | — | — |

### EXPLANATION OF PLATES

FIG. 1.—Agar gel precipitation reactions of tissue cell sap fractions with antiserum against tumour D8 cell sap.
D8TCS—Tumour D8 cell sap.
NLiCS—Normal liver cell sap.
NRS—Normal rat serum.
Anti-D8TCS abs—Rabbit antiserum against tumour D8 cell sap absorbed with normal liver cell sap (2·5 mg. protein/ml. antiserum) and normal rat serum (0·1 ml./ml. antiserum).

FIG. 2.—Agar gel precipitation reactions of tissue cell sap fractions with antiserum against tumour D14 cell sap.
D14/4–7—cell sap fractions from fourth to seventh generation of tumour D14 transfer.
NLi—Normal liver cell sap.
NRS—Normal rat serum.
Anti-D14—Rabbit antiserum against tumour D14 cell sap absorbed with normal liver cell sap (5 mg. protein/ml. antiserum) and normal rat serum (0·3 ml./ml. antiserum).

FIG. 3.—Agar gel precipitation reactions of deoxycholate solubilised microsomal fractions of tissue and rabbit antiserum against tumour D14 microsomes.
D8/13 and 17—Microsomal fractions from thirteenth and seventeenth generation of transplant of tumour D8.
D14/6 and 7—Microsomal fractions from sixth and seventh generation of transplant of tumour D14.
NLi—Normal liver microsomes.
Anti—D14 Rabbit antiserum against tumour D14 microsomes absorbed with normal liver microsomes (1·2 mg. protein/ml. antiserum) and normal rat serum (0·2 ml./ml. antiserum).

FIG. 4.—Agar gel precipitation reactions of tumour D8 and D14 cell sap fractions.
D8—Tumour D8 cell sap.
D14—Tumour D14 cell sap.
Anti-D8—Rabbit antiserum against tumour D8 cell sap absorbed with normal liver cell sap (2·5 mg. protein/ml. antiserum), normal rat serum (0·1 ml./ml. antiserum) and tumour D14 cell sap (1·2 mg. protein/ml. antiserum).
Anti-D14—Rabbit antiserum against tumour D14 cell sap absorbed with normal liver cell sap (2·5 mg. protein/ml. antiserum), normal rat serum (0·1 ml./ml. antiserum) and tumour D8 cell sap (1·8 mg. protein/ml. antiserum).

FIG. 5.—Agar gel precipitation reactions between tumour cell sap fractions and antiserum against tumour D14 cell sap.
D14—Tumour D14 cell sap.
D23—Tumour D23 cell sap.
NBRS—New born rat serum.
Anti-D14—Rabbit antiserum against tumour D14 cell sap absorbed with normal liver cell sap (2·5 mg. protein/ml. antiserum) and normal rat serum (0·1 ml./ml. antiserum).

FIG. 6.—Agar gel precipitation reactions between tissue cell sap fractions and antiserum against tumour D8 cell sap.
D8TCS—Tumour D8 cell sap.
Spleen CS—spleen cell sap.
Lung CS—Lung cell sap.
Anti-D8 TCS abs—Rabbit antiserum against tumour D8 cell sap absorbed with normal liver cell sap (2·5 mg. protein/ml. antiserum) and normal rat serum (0·1 ml./ml. antiserum).

1

2

3

Baldwin and Barker.

D8
anti D14
anti D8
D14

4

D 14
D 23
anti D14
NBRS
D14

5

D8 Tcs

Spleen cs

Anti D8Tcs abs

Lung cs

D8 Tcs

6

### DISCUSSION

These results demonstrate antigens in both the cell sap and microsomal fractions of transplanted rat hepatomas originally induced with 4-dimethylaminoazobenzene (DMAB) which are not detectable in normal rat liver or serum. As previously shown with primary hepatomas (Baldwin, 1965) the pattern of abnormal antigens is complex, tumour D8 exhibiting three, and tumour D14 one, cell sap antigens whilst both tumours contain four abnormal microsomal antigens. Furthermore, these antigens appear to be stable characteristics of each tumour, since no qualitative differences were detected during the limited period of tumour transplantation used in these studies.

The multiplicity of abnormal antigens detected contrasts with other observations (Deckers and Maisin, 1961; Abelev and Avenirova, 1960; Hirai *et al.*, 1963) where only a single new antigen was found in transplanted tumours induced with aminoazo dyes. Variations in experimental methods and perhaps more particularly differences in the potencies of antisera utilised may have contributed to these differences. Additionally, however, most other studies have used long-transplanted tumours and it is likely that deletion of antigen may have occurred on transplantation especially if there were isoantigenic differences between tumour and host. Thus, for example, Baldwin and Barker (1967) and Guelstein and Khramkova (1965) have shown that normal tissue antigens are deleted from experimental hepatomas even after only a few generations of transplantation in isogeneic hosts.

Whilst the abnormal microsomal antigens detected were common to both tumour D8 and D14, the new cell sap antigens of these two tumours were found to differ. Comparable differences in the antigenic composition of 2-acetylaminofluorene-induced rat hepatomas can be implied from demonstrations of specific immunofluorescent staining by rabbit antisera prepared against individual tumours (Hiramoto, Jurand, Bernecky and Pressman, 1963). However each of the cell sap antigens of D8 and D14 were detectable in other hepatomas induced by DMAB, occurring in various combinations in individual tumours (Table I). It is unlikely therefore that these antigens can be correlated with the tumour specific transplantation antigens demonstrated by the induction of resistance to hepatoma transplanted into isogeneic rats, since these have been shown to be individually distinct (Baldwin and Barker, 1965). This is further supported by the finding that pre-treatment of rats with either cell sap or microsome fractions of a transplanted hepatoma (D23) was without influence on the subsequent development of a challenge with the same tumour (unpublished observations).

Whilst the abnormal cell sap and microsomal antigens demonstrable in tumours D8 and D14 could not be detected in normal rat liver or serum, one D8 cell sap antigen was found in rat lung and spleen and three of the four microsomal antigens were identifiable in other normal rat tissues. Furthermore, the D14 cell sap antigen cross-reacted with a component of new born or foetal rat serum. None of the other abnormal tumour antigens could be found in normal tissues but this does not prove unequivocally that they are tumour specific since the possibility cannot be excluded that they occur in other normal or embryonic tissues. Whilst these observations further indicate that the antigens demonstrable by immunodiffusion methods are not related to the tumour specific transplantation antigens, they are nevertheless of considerable interest in that they probably reflect processes of dedifferentiation of liver during neoplastic transformation. Thus, for example, the cross-reaction of the D14 cell sap antigen, which was also detected in two out of thirteen other hepatomas, with an embryonic serum component compares with the finding of an embryonic serum $\alpha$ globulin in *o*-aminoazotoluene-induced mouse hepatomas (Abelev *et al.*, 1963). Also an embryonic $\alpha$ globulin has been detected in the serum of a patient with carcinoma of liver (Kithier, Houstek Masopust and Radl, 1966). The finding that several of the antigens in DMAB-induced hepatomas, whilst absent from liver, could be found in other normal tissues compares with the observation by Day (1965) that antisera against microsome fractions of 2-acetylaminofluorene-induced rat hepatomas could be shown by fluorescence microscopy to cross-react with rat kidney. Gold and Freedman (1965) also showed that an antigen demonstrable in human colonic carcinoma, whilst not detectable in normal adult tissues, could be found in a number of human embryonic tissues and was thus termed a carcinoembryonic antigen.

It would thus appear that neoplastic transformation induced in rat liver by DMAB is accompanied by a loss of antigens characteristic of the tissue of origin (Baldwin, 1964) together with the appearance of new antigens, some of which are demonstrable in other adult or embryonic tissues and others which are specific for the individual tumour (Baldwin and Barker, 1965). It still remains to be established whether these antigenic changes contribute to the functioning of the tumour cell or reflect significant causative processes during carcinogenesis.

### SUMMARY

1. Abnormal cell sap and microsomal antigens, not present in normal liver or adult rat serum, have been demonstrated using immunodiffusion methods in two rat hepatomas induced with 4-dimethylaminoazobenzene (DMAB). The microsomal antigens were common to both tumours whilst those in the cell sap differed. These cell sap antigens were all detectable however in differing combinations in other DMAB-induced hepatomas thus indicating that they were not tumour specific.

2. Certain of the new tumour cell sap and microsomal antigens could be detected in normal rat tissues other than liver or in foetal rat serum and these are considered to be a manifestation of processes of dedifferentiation during carcinogenesis.

This work was supported by a block grant from the British Empire Cancer Campaign for Research.

### REFERENCES

Abelev, G. I. and Avenirova, Z. A.—(1960) *Vop. Onkol.*, **6**, 57.

Abelev, G. I., Perova, S. D., Khramkova, N. I., Postnikova, Z. A. and Irlin, I. S.—(1963) *Transplantation*, **1**, 174.

Baldwin, R. W.—(1964) *Br. J. Cancer*. **18**, 285.—(1965) *Br. J. Cancer*, **19**, 894.

Baldwin, R. W. and Barker, C. R.—(1965) *Rep. Br. Emp. Cancer Campn*, **43**, 365.—(1967) *Nature, Lond.*, **214**, 292.

Day, E. D.—(1965) *Proc. Am. Ass. Cancer Res.*, **6**, 13.

Deckers, C.—(1964) ' Structure Antigénique de Tumeurs Expérimentales ', Brussels (Edition Arscia SA.).

Deckers, C. and Maisin, J.—(1961) *Rev. Belge. Path. Méd. exp.*, **28**, 477.

Gold, P. and Freedman, S. O.—(1965) *J. exp. Med.*, **122**, 467.
Guelstein, V. I. and Khramkova, N. I.—(1965) *Neoplasma*, **12**, 251.
Hirai, H., Taga, H., Isaka, H., Satoh, H. and Warabioka, K.—(1963) *Gann*, **54**, 177.
Hiramoto, R., Jurand, J., Bernecky, J. and Pressman, D.—(1963) *Cancer Res.*, **23**, 109.
Kithier, K., Houstek, J., Masopust, J. and Radl, J.—(1966) *Nature, Lond.*, **212**, 414.
Lowry, O. H., Rosebrough, N. J., Farr, A. L. and Randall, R. J.—(1951) *J. biol. Chem.*, **193**, 265.